# A Multi-scale Analysis of Influenza A Virus Fitness Trade-offs due to Temperature-dependent Virus Persistence

**DOI:** 10.1371/journal.pcbi.1002989

**Published:** 2013-03-21

**Authors:** Andreas Handel, Justin Brown, David Stallknecht, Pejman Rohani

**Affiliations:** 1 Department of Epidemiology and Biostatistics, College of Public Health, University of Georgia, Athens, Georgia, United States of America; 2 Department of Population Health, College of Veterinary Medicine, The University of Georgia, Athens, Georgia, United States of America; 3 Department of Ecology and Evolutionary Biology, University of Michigan, Ann Arbor, Michigan, United States of America; 4 Center for the Study of Complex Systems, University of Michigan, Ann Arbor, Michigan, United States of America; 5 Fogarty International Center, National Institutes of Health, Bethesda, Maryland, United States of America; Imperial College London, United Kingdom

## Abstract

Successful replication within an infected host and successful transmission between hosts are key to the continued spread of most pathogens. Competing selection pressures exerted at these different scales can lead to evolutionary trade-offs between the determinants of fitness within and between hosts. Here, we examine such a trade-off in the context of influenza A viruses and the differential pressures exerted by temperature-dependent virus persistence. For a panel of avian influenza A virus strains, we find evidence for a trade-off between the persistence at high versus low temperatures. Combining a within-host model of influenza infection dynamics with a between-host transmission model, we study how such a trade-off affects virus fitness on the host population level. We show that conclusions regarding overall fitness are affected by the type of link assumed between the within- and between-host levels and the main route of transmission (direct or environmental). The relative importance of virulence and immune response mediated virus clearance are also found to influence the fitness impacts of virus persistence at low versus high temperatures. Based on our results, we predict that if transmission occurs mainly directly and scales linearly with virus load, and virulence or immune responses are negligible, the evolutionary pressure for influenza viruses to evolve toward good persistence at high within-host temperatures dominates. For all other scenarios, influenza viruses with good environmental persistence at low temperatures seem to be favored.

## Author Summary

It has recently been suggested that for avian influenza viruses, prolonged persistence in the environment plays an important role in the transmission between birds. In such situations, influenza virus strains may face a trade-off: they need to persist well in the environment at low temperatures, but they also need to do well inside an infected bird at higher temperatures. Here, we analyze how potential trade-offs on these two scales interact to determine overall fitness of the virus. We find that the link between infection dynamics within a host and virus shedding and transmission is crucial in determining the relative advantage of good low-temperature versus high-temperature persistence. We also find that the role of virus-induced mortality, the immune response and the route of transmission affect the balance between optimal low-temperature and high-temperature persistence.

## Introduction

Influenza A viruses infect both humans and animals, causing frequent outbreaks [1], [2]. In humans, the infection can be life-threatening for individuals with weak immune systems, leading to an estimated annual worldwide mortality burden of 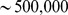 [3], [4]. Due to its zoonotic nature, and frequent spillover from wild and livestock populations, eradication of the virus is virtually impossible [1], [5]. Further, the danger that a novel influenza strain with high virulence and pandemic potential will start to spread in the human population is always present [6]–[8]. The 2009 H1N1 pandemic demonstrated that the emergence of novel pandemic strains is still largely unpredictable. Improvement of our surveillance, prediction and control capabilities requires that we obtain a better understanding of the whole transmission cycle of the virus and the mechanisms governing the complex processes of infection and spread.

One useful approach for studying the whole infection and transmission process is through the use of multiscale studies, wich have seen increased general development and use in recent years (see e.g. [9], [10] for reviews and [11] for a recent application to influenza). A multiscale approach allows one to address the question of how different selection pressures on the within- and between-host levels interact to impact overall fitness. This is important if we want to better understand and predict the infection and transmission dynamics and evolution of the virus.

Here, we use such a multiscale framework and focus on one specific aspect, namely evolutionary pressures shaped by temperature-dependent virus persistence. The importance of temperature on influenza virus fitness is well established. For instance, the attenuated live influenza vaccine is cold-adapted, which leads to reduced fitness in human hosts, making it safe for vaccination purposes [12], [13]. Temperature has also been shown to impact within-host dynamics and transmission in laboratory studies [14], [15]. Recent theoretical and experimental evidence suggests that persistence in the environment is an important factor of transmission for avian influenza [16]–[24]. Transmission through an environmental stage (e.g. long-lasting droplets, fomites) seems to also play a role for influenza transmission in humans [25]–[29]. Since temperatures in the environment and within a host can be markedly different, it is possible that the virus faces a trade-off: It can either optimize its ability to persist within a host, or optimize its ability to persist outside a host. It is well known that the decay rate of most viruses depends on temperature, with faster virion decay occurring at higher temperature [30]–[32]. Interestingly, recent data [33] suggest that temperature-dependent decay rates differ between influenza strains. Some strains are very stable at environmental temperatures (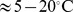) but rapidly decay at higher within-host temperatures (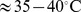), while others persist less well at low temperatures but also have a less rapid decay as temperature increases [33]. These data suggest that some virus strains might optimize persistence within a host, while others might optimize persistence outside a host, with a possible trade-off between the two. This in turn can affect both within-host and between-host dynamics. The dynamics on these two levels interact to determine overall fitness. (Note that the data presented in [33] – which we will analyze below – is for different HA-NA serotypes. However, the phenomenon of temperature-dependent decay we discuss is not specific to distinct serotypes. We will therefore use the generic term “strain” throughout this study).

To analyze the impact that such a temperature-dependent trade-off can have on virus fitness, we build a multi-scale model that embeds a within-host infection process within a population transmission framework. A number of theoretical studies have previously considered trade-offs between environmental persistence and within-host performance, see e.g. [34]–[38]. Those studies considered generic trade-offs and models without direct relation to a specific pathogen or fitting to data. A few notable studies that involved data looked at environmental survival and virulence of human pathogens [39] and environmental survival and growth in phages [40]. Here, we focus on avian influenza A and combine experimental data with models to explicitly consider temperature-dependent virus decay as the mediator of trade-offs. We find that for direct transmission scenarios, viruses with long within-host persistence perform overall best. For environmental transmission scenarios, the balance was shifted toward viruses with good environmental persistence. This was especially true if shedding or infection rates were assumed to be proportional to the logarithm of the virus load. We further show that the addition of an immune response or pathogen virulence reduced the importance of differences in the within-host decay rate between strains, and lead to an increased importance of good environmental persistence.

## Models

### The within-host model

**Figure 1:**
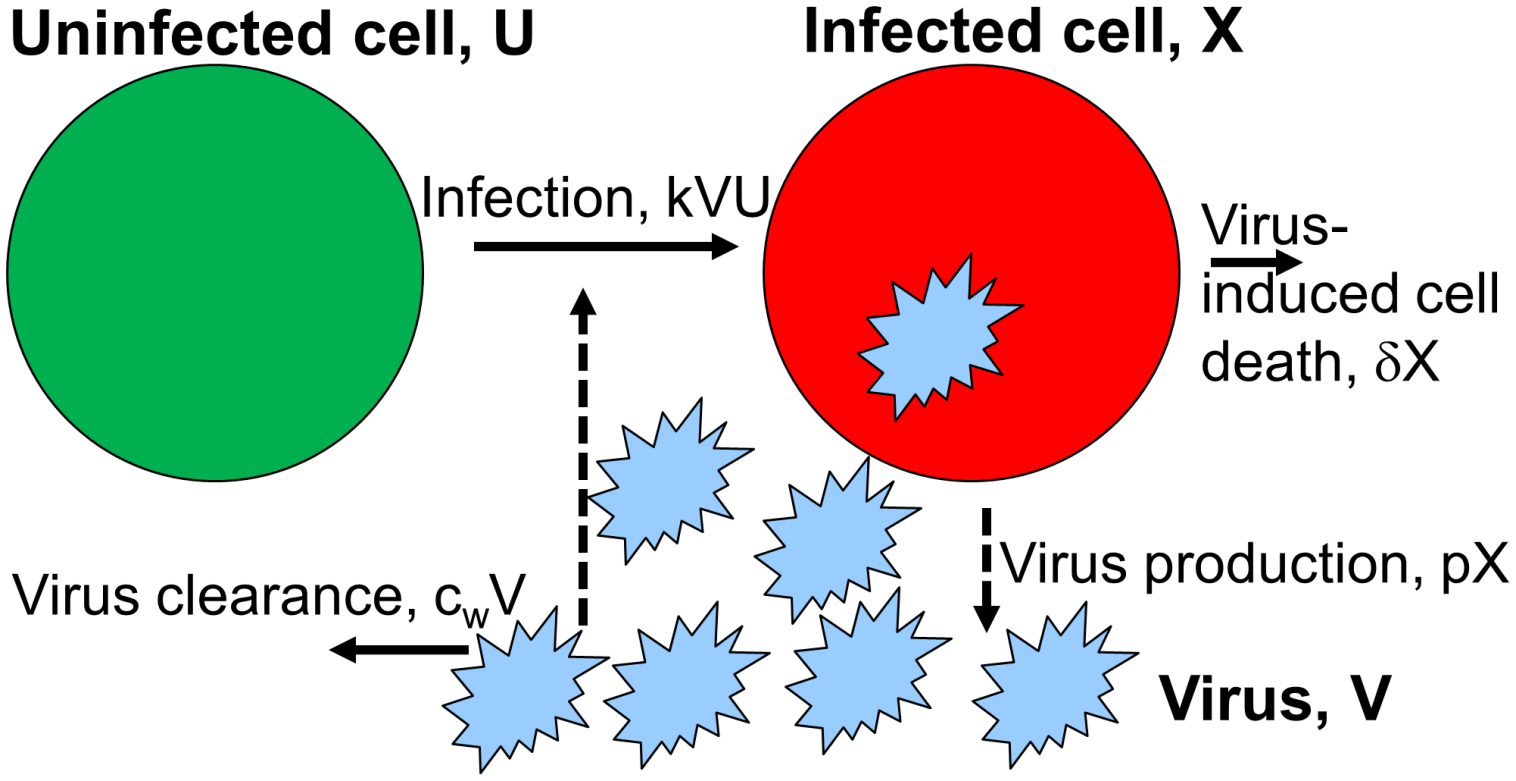
Flow diagram for the within-host model. 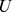
, 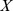, and 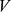 are the variables describing uninfected cells, infected cells, and infectious virus. Uninfected cells become infected at rate 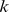, infected cells produce virus at rate 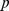 and die at rate 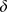. Virus decays at rate 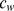. Solid lines indicate physical flows, dashed lines indicate interactions.

We consider a simple model for an acute viral infection. These types of models have been used in several recent analyses of influenza A virus within-host infection dynamics (see e.g. [41], [42] for reviews). Our model tracks uninfected cells, 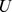, infected cells, 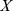, and infectious virus, 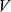. Cells become infected at rate 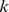, infected cells produce virus at rate 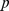 and die at rate 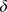. Infectious virus decays at rate 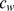. The model equations are given by
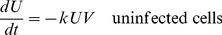
(1)

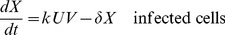
(2)

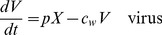
(3)The model is illustrated in figure 1, table 1 summarizes the model variables and parameters.

**Table 1:** Initial conditions and parameter values for the within-host model.

symbol	meaning	values	comment
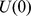	target cells	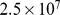	based on [71]
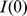	initial number of infected cells	0	assumed
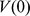	inoculum dose	1 EID_50_/mL	assumed
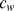	virus clearance rate	2.78 per day	fixed, see text
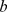	infection rate	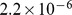 mL/EID_50_ per day	fitted
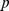	production rate of virions	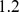 EID_50_/mL per day	fitted
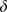	death rate of infected cell	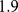 per day	fitted

Initial conditions and parameter values for the within-host model. 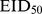 = 50% Egg Infectious Dose.

This simple model can describe most data for influenza virus infections rather well [41], [42]. After an initial rise in virus load, uninfected target cells become depleted, leading to a subsequent virus decline and resolution of the infection. This so-called target-cell limited model is basically equivalent to a simple epidemic model, which produces a single infectious disease outbreak in a susceptible population. However, it is also known that influenza infections stimulate an immune response, which likely plays some role in viral clearance, though the exact contributions of various components of the immune response to virus clearance are still not fully understood. We consider an alternative model with an immune response in the supplementary materials.

### The between-host model

To describe influenza transmission dynamics on the between-host level, we use a framework that takes into account both direct and environmental transmission routes, as has been recently advocated [16], [17]. Similar models – not specific to influenza – that explicitly include an environmental stage have been designed and analyzed previously [35], [36], [38], [43]–[46].

**Figure 2:**
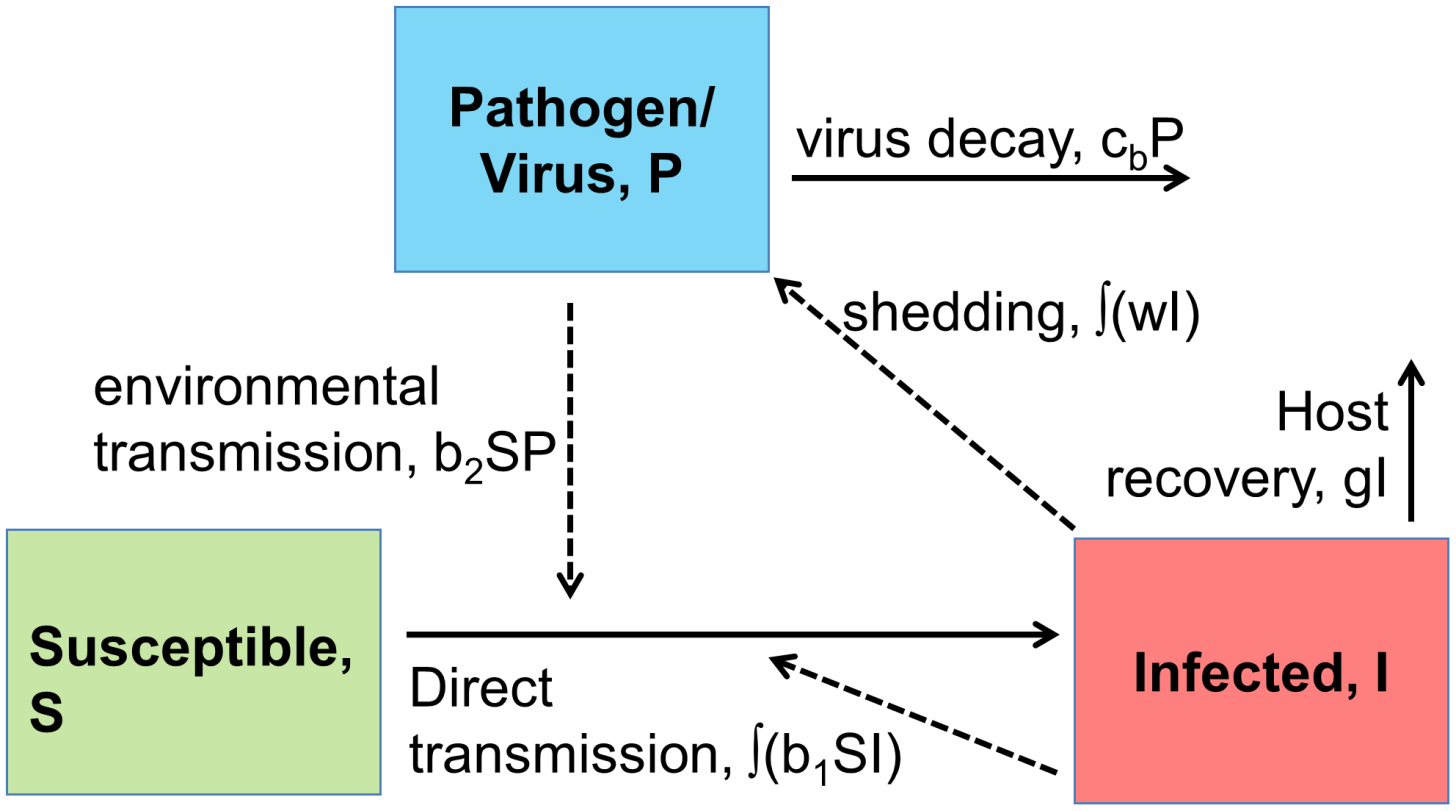
Flow diagram for the between-host model. 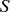
, 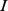 and 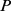 are the variables describing susceptible hosts, infected hosts, and pathogen (i.e. virus) in the environment. Transmission can occur directly between uninfected and infected hosts at rate 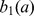 and through contact of uninfected hosts with virus in the environment at rate 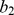. Infected hosts shed virus into the environment at rate 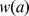, and recover (and are assumed to become immune to re-infection) at rate 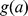. Virus in the environment decays at rate 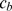. Note that the parameters 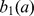, 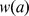 and 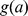, i.e. the rate of transmission between hosts, the rate of shedding and the rate of recovery all depend on the time since infection. Solid lines indicate physical flows, dashed lines indicate interactions.

We use coupled partial differential equations to allow for explicit tracking of the age of infection within the infected population. This allows for convenient linking of the within- and between-host scales as described below. The model is shown and explained in figure 2 and legend, table 2 summarizes model quantities. The model equations are given by
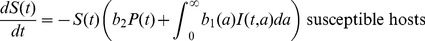
(4)

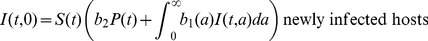
(5)

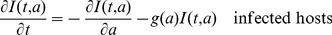
(6)

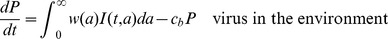
(7)Time 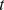 indicates the usual “system time”, while 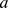 indicates the time since infection of a host. The parameters 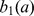, 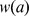 and 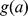, i.e. the rate of transmission between hosts, the rate of shedding and the rate of recovery all depend on the time since infection. We will choose specific forms for those parameters in the next section.

**Table 2:** Parameters for the between-host model.

symbol	meaning
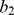	environmental infection rate
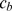	virus decay rate in the environment
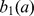	direct transmission rate^*^
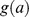	rate of recovery^*^
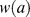	rate of shedding^*^

Parameters for the between-host model. Parameters marked with ^*^ depend on time 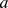 since start of infection. Specific choices for these parameters are described in the text. Note that we do not make use of specific numeric values for any of these parameters, therefore none are given.

Note that we do not actually simulate the between-host dynamical process. The reason for specifying the between-host model is to compute the basic reproductive number, 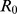, which is our measure of between-host fitness (see next section). Analysis of other fitness measures that would require simulating the between-host dynamical process (e.g. probability of extinction over multiple outbreaks) is a suitable subject of future studies but will not be considered here.

### Defining fitness and connecting the two scales

Our main quantity of interest is fitness of the virus at the host population level. One way to quantify fitness is through the basic reproductive number, 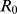, which is defined as the expected number of new infections caused by one infected host in a fully susceptible population [47]–[49]. For our model, one can split 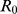 into two components, namely direct transmission from host to host (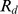), and indirect transmission through the environmental route (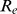), such that 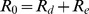 [17], [36]. For direct transmission, we have
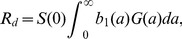
(8)where 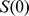 is the susceptible population at time 0, 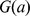 is fraction of hosts that are still infectious at time 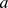 after infection started, and 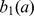 denotes the rate at which an infectious individual at infection age 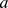 infects new individuals. If we assume that all infected hosts are infectious for a fixed duration, 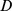, and non-infectious afterwards, we can write
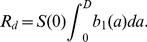
(9)Mathematically, this corresponds to choosing the proportion of host infectious after time 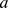, 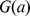, as a Heaviside function, and the recovery rate, 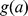, in the between-host model equations as a Dirac delta-function. While the infectious period could end either due to resolution of the infection (recovery) or host death, for the low pathogenic influenza strains we consider here, mortality is negligible [50]–[52]. Therefore, for the main part of this study, the end of the infectious period should be interpreted biologically as recovery. In the supplementary materials we briefly consider virus-associated mortality (i.e. virulence) and how it might alter the results presented in the main part of the manuscript.

We can define the duration of infectiousness 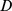 in terms of the within-host model, as the time from the start until the end of the infection, which we define as the time virus levels drop below a given level, 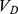 (in our simulations chosen to be one virion). Mathematically, this can be written as
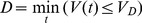
(10)The rate at which direct transmission between hosts occurs, 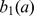, also likely depends on the within-host dynamics. One possible assumption is that 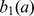 is directly proportional to virus load:
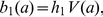
(11)where 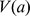 is the virus load at time 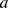 after infection and 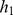 is some constant of proportionality. This assumption corresponds to the “flu like infection regime” in [53], and seems to be a reasonable approximation [54]–[57]. Defining
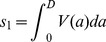
(12)as the total infectious virus during the infection (area under the curve), and substituting equations (12) and (11) into (9), we obtain as expression for the directly transmitted virus fitness
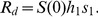
(13)While a linear relationship between transmission and virus load, as described by equation (12), is plausible, it is certainly not the only possibility. For instance, we previously showed that a sigmoid function of the form
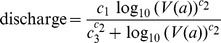
(14)provides a good description of the total amount of nasal discharge as function of virus load for human influenza A infections [58]. Here, the coefficients 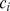 describe the shape of the sigmoid curve. While the hosts in the present study are ducks, not humans, we submit that representing the total amount of discharge by a sigmoid curve makes inherent biological sense for any host. Multiplying virus load by the amount of discharge and integrating over the duration of infection gives
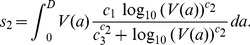
(15)For our numerical analysis below, we set 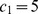, 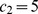, 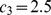, which are values close to those previously determined by fit of this sigmoid curve to shedding data for humans [58]. The exact values for those coefficients matter little for the results we present in this study. Using the equation for 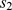 instead of the equation for 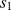 in equation (13) is an alternative for linking within-host dynamics to between-host fitness. Another plausible scenario is one where the rate of transmission is proportional to the logarithm of the virus load, giving
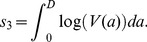
(16)We can use this expression in equation (13) instead of 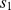. Such a logarithmic dependence of transmission on virus load makes especially good sense given that 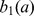 and therefore 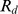 are a measure for the number of new infections produced, which not only includes the shedding and transmission process, but also includes the probability that a subsequent infection in a new host is started. A logarithmic dependence between pathogen dose and the probability of infection occurring appears to be common [53], [59]–[63]. Since it is not known which assumption for the link from within-host virus load to between-host transmission is most applicable to the host-pathogen system we study here, we will investigate all three possible functions 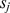 (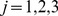) and their impact on host population level fitness as measured by 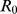.

The environmental transmission component of fitness, 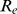, can be linked to the within-host model in the same way as just described for the direct component, 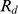. Specifically, we can write
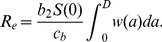
(17)The rate of viral shedding into the environment, 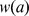, again depends on the within-host dynamics. If we assume that 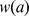 depends on the within-host virus load in the same way as the direct transmission rate 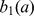, we obtain
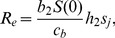
(18)where the terms 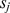 represent the different link functions described in equations (12), (15) and (16), and 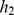 is another constant of proportionality. Table 3 summarizes the important quantities we introduced in this section.

**Table 3:** Summary of quantities linking the within-host and between-host scales.

symbol	meaning
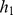	constant of proportionality connecting virus load and direct transmission rate
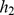	constant of proportionality connecting virus load and environmental transmission rate
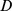	duration of infectiousness, obtained from the within-host model (equation 10)
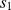	link-function to connect virus load with transmission, assuming linear relation (equation 12)
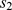	link-function to connect virus load with transmission, assuming linear relation modified by total shedding (equation 15)
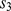	link-function to connect virus load with transmission, assuming logarithmic relation (equation 16)

### Model implementation

All statistical analyses and simulations were done in the R programming environment [64]. The scripts are available from the corresponding author's webpage (http://ahandel.myweb.uga.edu/resources.htm).

## Results

Both the within- and between-host models contain a term for virus decay, namely 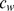 and 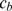. It is obvious that to maximize fitness, the virus should minimize both 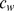 and 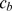, i.e. it should be able to persist well both outside the host (in the air or on fomites for humans, in water for avian species) and inside the host. However, as we show below, there seem to be trade-offs between the ability to persist at low versus high temperatures. While higher temperatures lead to faster decay for all strains, some strains are better at persisting in the environment at low temperatures (low 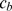), but this comes at the cost of rapid decay inside a host at higher temperatures (high 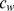). In contrast, other strains seem to persist less well at low temperatures, but as temperature increases, their decay rate increases less rapidly, making them more stable at higher temperatures. Given this potential trade-off between 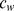 and 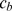, we analyze how within- and between-host levels interact to determine overall virus fitness on the host population level as measured by 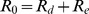. Within-host decay, 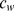, affects within-host viral dynamics and thereby, through the link-functions 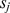, both the direct and environmental fitness components 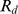 and 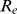 (equations 13 and 18). The between-host decay term, 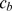, only affects the environmental fitness component, 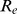. Therefore, we expect that depending on transmission route and link functions, the impact of good low- versus high-temperature persistence on fitness can change. We will show how this plays out in the following.

### Temperature dependence of viral decay

**Figure 3:**
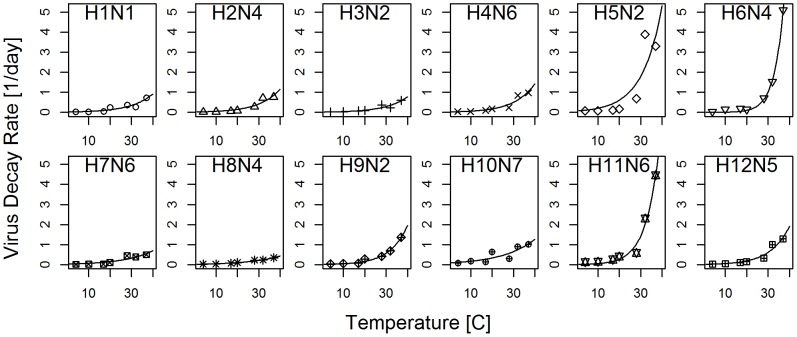
Decay rate for 12 different influenza strains as function of temperature. Symbols show data, lines show best fit of an exponential function. Virus decay for all strains was measured at the indicated temperature, a pH of 7.2, and salinity of 0. Decay for each strain was measured once for these specific conditions. See [33] for more experimental details.

In a recent study [33] we found that for a panel of different avian influenza A strains, the decay rate of infectious virus varies as a function of temperature. We can quantify the virus decay rate, 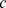, as a function of temperature, 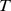. The data suggest that a simple exponential function of the form 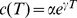 fits each strain well. Figure 3 shows the data and best-fit exponential curves, with the estimated values for 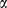 and 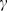 provided in table 4. The simple equation 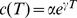 allows us to compute decay rates at a within-host temperature of around 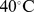 corresponding to the body temperature of a duck [15], [65] and at a between-host environmental temperature assumed to be cold lake water at around 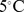. Those quantities correspond to 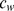 and 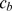 in our within-host and between-host models. Table 4 lists their values for the different strains.

**Table 4:** Best fit values for the different influenza strains.

strain, (group)	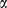 , (rank)	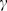 , (rank)	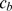 , (rank)	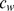 , (rank)
H1N1, (1)	0.019, (5)	0.097, (4)	0.031, (5)	0.914, (4)
H2N4, (1)	0.02, (7)	0.101, (6)	0.033, (6)	1.147, (5)
H3N2, (2)	0.015, (4)	0.098, (5)	0.025, (3)	0.772, (3)
H4N6, (2)	0.021, (8)	0.105, (7)	0.036, (8)	1.427, (7)
H5N2, (1)	0.07, (11)	0.108, (8)	0.121, (11)	5.316, (10)
H6N4, (1)	0.001, (1)	0.23, (12)	0.003, (1)	10.145, (12)
H7N6, (2)	0.034, (10)	0.076, (3)	0.049, (10)	0.697, (2)
H8N4, (1)	0.026, (9)	0.071, (2)	0.037, (9)	0.438, (1)
H9N2, (1)	0.014, (3)	0.123, (10)	0.027, (4)	1.948, (9)
H10N7, (2)	0.09, (12)	0.066, (1)	0.125, (12)	1.263, (6)
H11N6, (1)	0.011, (2)	0.163, (11)	0.025, (2)	7.334, (11)
H12N5, (1)	0.02, (6)	0.114, (9)	0.035, (7)	1.919, (8)

Best fit values for the different strains fitted to the function 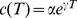. Parameter 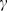 is in units of 1/degree Celsius, 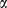 is in units of 1/day. Also shown are decay rates (units of 1/day) for each strain at 5 (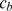) and 40 (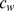) degrees Celsius. Numbers in parentheses following each strain indicate the genotype group (see main text). Numbers in parentheses following the other values indicate the rank of this value for each strain (with rank 1 given to the strain with the lowest value, corresponding to better persistence.)

**Figure 4:**
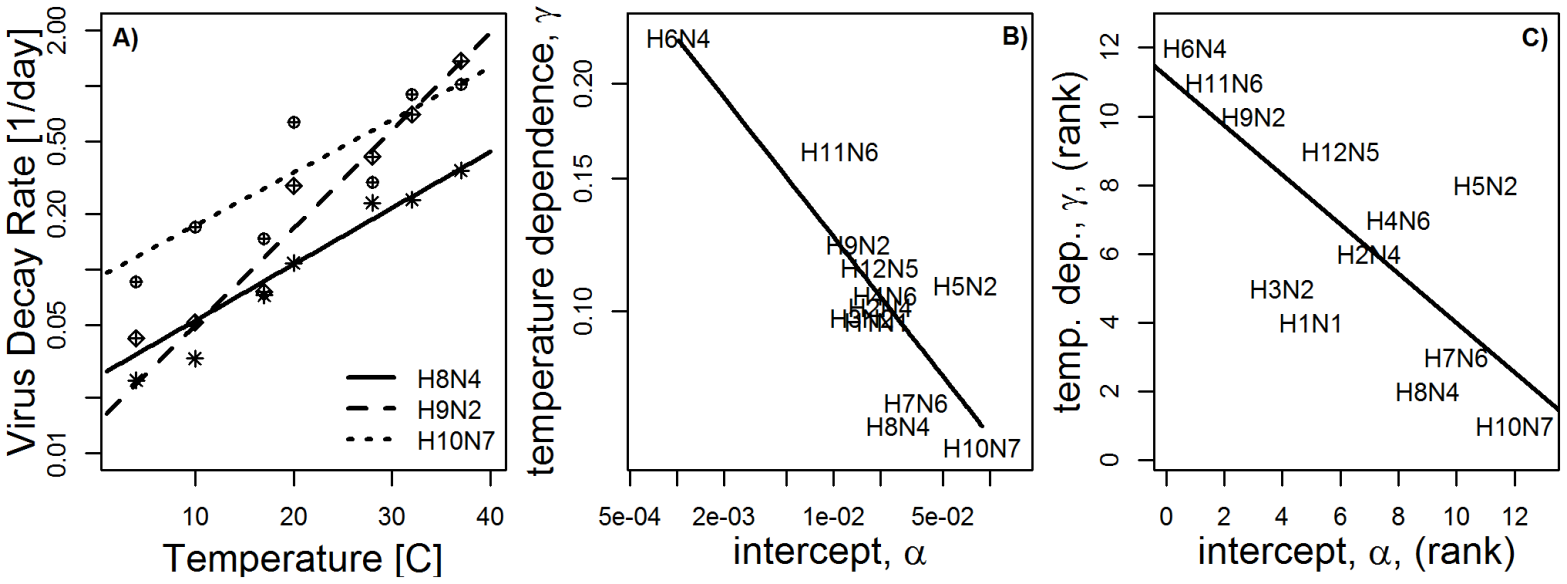
Temperature trade-off between strains. A) Decay rates for H8N4, H9N2 and H10N7, plotted on a log scale to illustrate the cross-over of decay rates. B) absolute values of 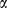 and 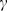 for all strains, (note the log scale). C) Ranks of these parameters. Also plotted in each figure are regression lines.

Figure 3 and table 4 suggest that while some strains have a relatively low (e.g. H3N2) or high (e.g. H5N2) decay rate irrespective of temperature, others appear to specialize. Some strains (e.g. H6N4, H11N6) decay relatively slowly at low temperatures but persist poorly at high temperatures, while others (e.g. H8N4, H7N6) do relatively better at high versus low temperature. Thus, some strains are able to persist for a long time at low temperatures, but as temperature increases, their rate of decay also rapidly increases. In contrast, other strains are not able to persist for quite as long at low temperatures, but increases in temperature leads to a slower rise in atrophy. As we illustrate in figure 4A, this can lead to a cross-over in decay rates as function of temperature. In figure 4B, we regress the strain-specific values for the intercept of the decay rate curve, 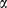, (quantifying virus persistence at low temperature, specifically at 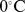) against the value for the temperature-dependence of the decay rate, 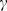, (quantifying virus persistence at high temperature). In figure 4C, we provide the same information, but for the rank of those parameters. These plots demonstrate a negative correlation between persistence at low and high temperatures. Since the center panel indicates a linear relation for the logarithm of 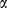 and 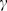, we fitted a regression line 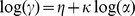 to the data. We find for the regression fit 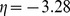, 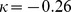 (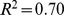, 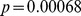). Similarly, computing a correlation coefficient for the rank-transformed data, we find a negative correlation of 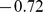 (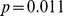).

The analysis of this dataset can be taken as suggestion for the presence of a trade-off between stability at low and high temperatures – at least for the panel of strains we investigated here. Since this is a small sample of strains, we do not want to over-emphasize the finding. However it seemed real and interesting enough to ask the questio: “How would such a potential trade-off lead to interactions on the within-host and between-host levels and affect overall virus fitness?”. We address this question in the remainder of the paper.

As a potentially interesting side question – not further considered in the remainder of this paper – we wondered whether there are systematic differences between strains belonging to different groups. Based on amino acid differences, strains with different HA types can be clustered into two groups, as indicated in Table 4 (see e.g. [66]–[68]). We were curious to see if systematic differences in the decay behavior between the two groups could be observed. However, statistical tests applied to both the absolute and rank-transformed values of 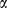 and 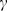 did not identify significant differences between groups, suggesting that – based on the available data – differences in HA sequences between the two groups do not express themselves phenotypically as differences in temperature-dependent decay characteristics.

### Fitting the within-host model

**Figure 5:**
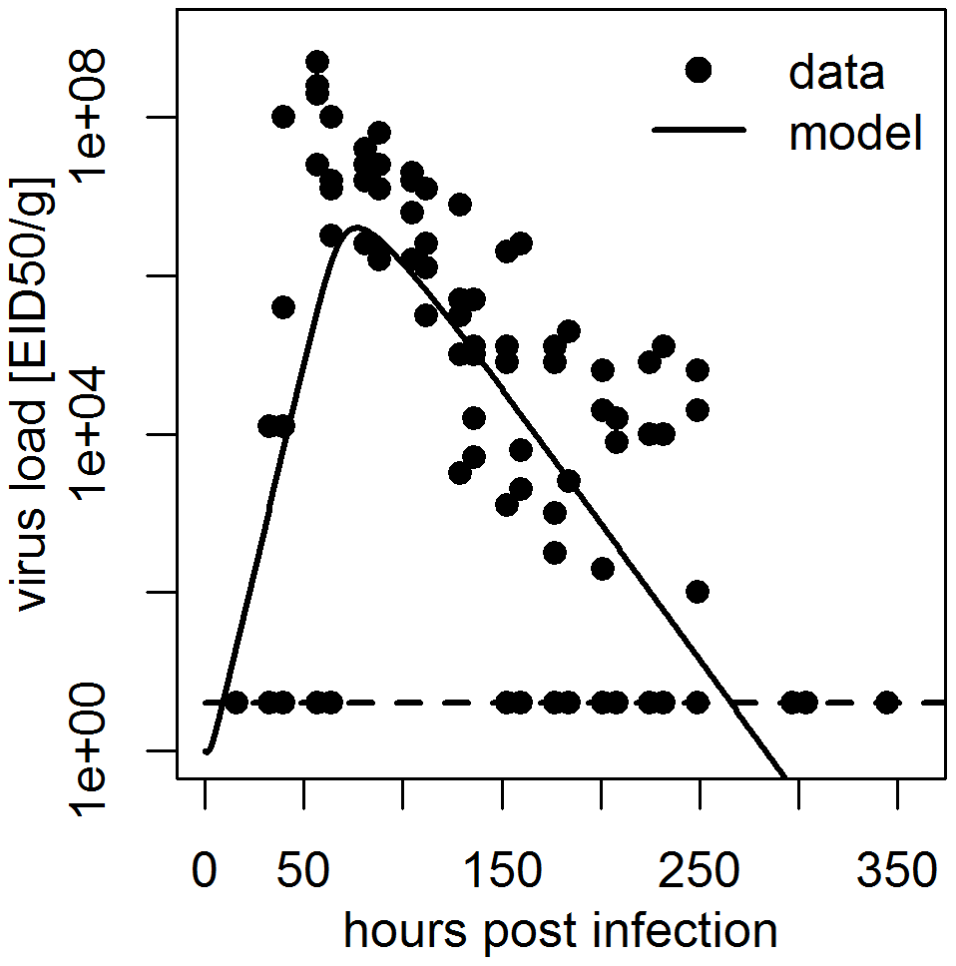
Best fit of within-host model to fecal virus load from influenza infections of mallards (Anas Platyrhynchos). The limit of detection for the virus load was 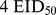 (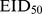 = 50% Egg Infectious Doses) and is indicated by the dashed horizontal line. See [69] for more details on the experiments and data. Fitting was done using a least squares approach for the logarithm of the virus load, corresponding to the assumption of log-normally distributed errors [89]. For data at the limit of detection (i.e. left-censored data), differences between model and data were accounted for if the model was above the data point, but not if the model took on any value below the limit of detection [75].

To simulate a within-host infection, we need to specify parameter values for the within-host model. While parameter estimates are available for influenza infections in humans and mice [41], [42], they have not been previously estimated for ducks. We therefore fitted the model to recent data from influenza infections with H3N8 in mallards (*Anas platyrhynchos*) [69]. This virus strain was not used in the decay experiments shown in table 4, therefore, we do not have a direct estimate for the within-host clearance rate, 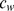. The straightforward approach would be to obtain 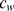 together with the other parameters by fitting to the data, but this approach is problematic. As has been shown previously, it is impossible to use the within-host model (equations 1–3) to accurately estimate both 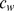 and death rate of infected cells, 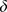, from virus titer data alone [42], . Because of this, we instead set 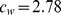 per day, which is the mean value of 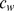 for the 12 strains reported in table 4. We also tried to fit 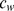, and as expected, the fit did not improve and 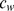 could not be properly estimated. To perform the fit, we assume that the infection was started by a 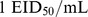 (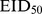 is the viral dose that results in a 50% chance of infecting an embryonated egg, assumed to correspond to 1 infectious virion) and that the initial number of uninfected target cells is 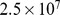 [71] (while this estimate is for chickens rather than ducks, the exact value is not qualitatively important: changes in the target cell numbers only re-scale the model parameter 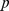 and otherwise produce the same dynamics). In figure 5, we show the best fit to the data, with parameter values presented in Table 1. We want to point out that while these parameter estimates are useful and accurate enough for the purpose of our study, they come with caveats. Most importantly, estimates are based on the validity of the model used. A model that does not include an immune response is likely an over-simplification, albeit a necessary one since adding additional immune response components and trying to fit such a model to virus load data only would lead to over-fitting. See e.g. [41], [42] and references therein for further discussions of this and related points concerning fitting influenza data to models.

### Determining between-host fitness

For each strain listed in table 4, we can use 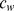 and the parameters determined in the previous section and simulate the within-host infection dynamics. This allows us to numerically determine the duration of infection, 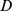, and the total virus load, which in turn specifies the different link functions, 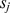. We also have estimates for 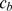 for each strain. To determine fitness as measured by 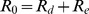, we also need to know the population size 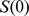, and several constants of proportionality, namely 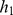 and 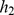 describing the linkage between within-host virus load and shedding and infection rates, and the environmental transmission rate, 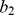. Those quantities are not well known and will likely differ for different environments. Therefore, absolute values of 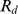 and 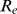 are hard to estimate. However, for any strain 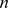 we can consider its fitness relative to some reference strain, 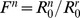. If we make the assumption that for a given scenario, 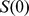, 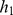, 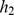 and 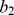 do not differ between strains; and consider the two extreme cases of either only direct (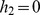) or only environmental (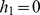) transmission, relative fitness for strain 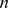 and link-function 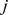 (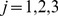), relative to some reference strain, 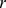, is given by
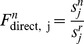
(19)for direct transmission and
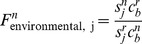
(20)for indirect, environmental transmission. As expected, if we consider only direct transmission (equation 19), the ability of the virus to persist at low temperatures (low 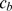) does not impact its fitness and therefore the strain that optimizes persistence at high temperatures (low 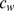) and therefore optimizes within-host dynamics (large 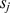) performs best. In the presence of environmental transmission (equation 20), fitness is influenced by persistence both inside the host (low 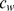, leading to high 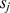) and in the environment (low 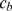).

The two cases, environmental only and direct only transmission represent extremes in terms of potential trade-offs. For direct transmission alone, there is no trade-off; optimizing within-host fitness is always the best strategy. The environmental transmission only scenario represents the case where the importance of the environmental stage is as large as it can possibly be. Mixture of the two transmission routes leads to values with intermediate importance of environmental persistence. While it is certainly possible to consider the general case with both direct and environmental transmission and compute absolute and relative fitness values, this would require making rather arbitrary assumptions about values for some of the unknown parameters of proportionality. Since considering such a general mixed transmission scenario would not add much beyond the results for the two simpler extreme cases, we focus on these two extreme cases in the following.

**Figure 6:**
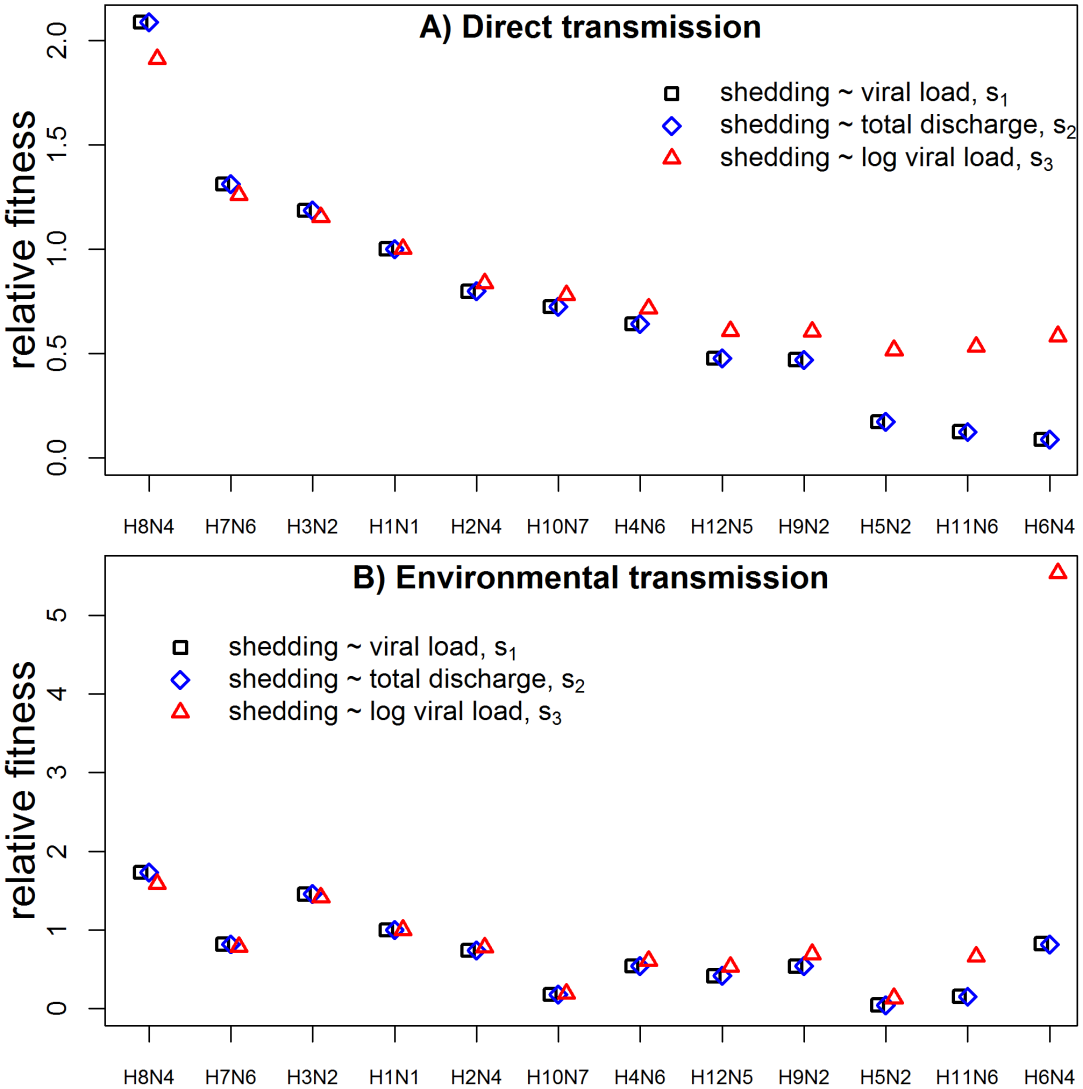
Relative Fitness for the 12 influenza strains. A) direct transmission (equation 19) and B) environmental transmission (equation 20) scenarios. We plot fitness for the three different link functions, 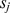, between within-host virus load and transmission/shedding described in the model section, i.e. 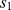, 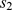 and 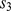 given by equations (12), (15) and (16). Strains are sorted according to within-host fitness, with H8N4 having the best within-host fitness (i.e. lowest value of 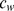, see Table 4). We arbitrarily chose H1N1 as the reference strain, which therefore has a relative fitness of 1.

In figure 6, we show the relative fitness of the 12 different strains, for exclusively direct or environmental transmission scenarios. We plot relative fitness for the three different link-functions between within-host virus load and transmission/shedding described above (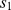, 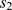 and 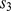 given by equations (12), (15) and (16)). Strains are sorted according to within-host performance (i.e. with increasing values of 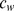). We arbitrarily chose H1N1 as the reference strain, which therefore always has a fitness of 1. As expected, for direct transmission (figure 6A), better within host persistence at high temperatures leads overall to higher fitness. Results differ little between the link function based on the simple linear assumption, 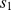, and the additional inclusion of total discharge, 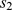. However, assuming that the amount of shedding is proportional to the logarithm of virus load, 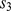, reduces the relative importance of within-host dynamics. Put another way, since 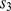 “counts” 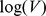 instead of 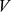, the fitness impacts of differences in within-host virus load between strains are diminished and, consequently, the relative fitness advantage of strains with high within-host persistence is reduced. This therefore increases the relative fitness of the strains with high 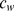. In fact, for the three strains with the lowest within-host fitness (H5N2, H11N6 and H6N4), the somewhat reduced within-host fitness due to higher 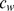 leads to lower virus load but a longer duration of infection, and because virus load factors into shedding only in a logarithmic fashion, a longer duration of infection leads to a slightly increased fitness despite higher 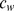. See also the next section for another appearance of this phenomenon. Note that it is unclear how biologically reasonable sustained within-host virus load (i.e. a long duration of infection) is. In most immunocompentent hosts, the immune response usually clears influenza relatively rapidly [72]–[77]. In the supplementary materials, we investigate an extended within-host model which includes an antibody-mediated immune response.

Not surprisingly, for the environmental transmission scenario (figure 6B), the trend of higher overall fitness for the strains with better within-host persistence is less pronounced. For instance, the H7N6 strain is the second fittest strain for the direct transmission scenario, but is surpassed in fitness for the environmental transmission scenario by several other strains with better low-temperature persistence. Again, results for the different link functions are rather similar. The one outlier is H6N4, which has the best low-temperature and worst high-temperature persistence. For this strain and link function 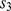, the reduction in relative importance of the high-temperature within-host dynamics compared to the low-temperature between-host persistence strongly increases this strain's relative fitness (see top left corner of figure 6B).

### General trade-off for viral decay

**Figure 7:**
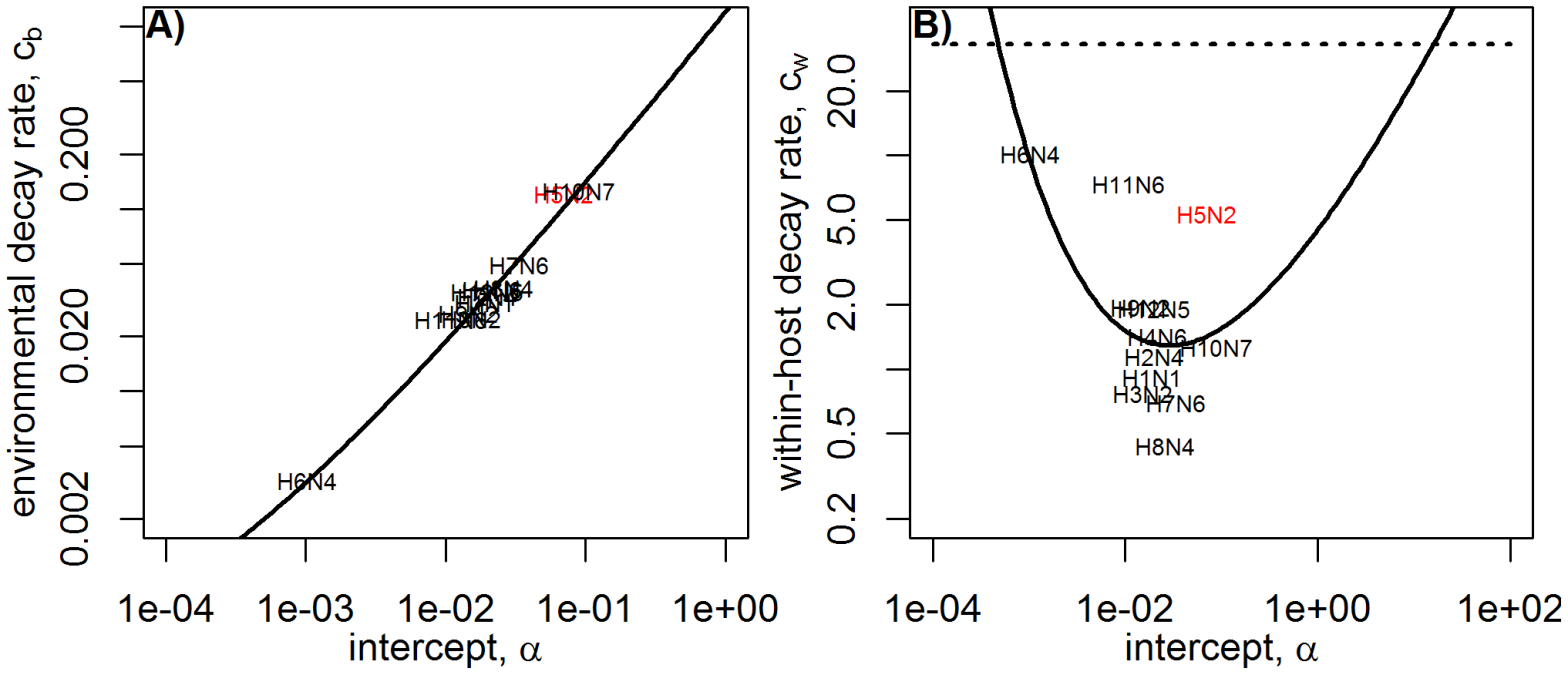
Virus decay rate at different temperatures. A) environmental, between-host temperature, 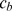, and B) within-host temperature, 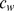, as a function of 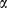 (decay rate at 0 degrees Celsius). Solid lines are theoretical values obtained by choosing a value of 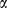 and computing the corresponding value for 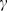 from the regression equation 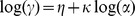, where the values for 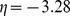 and 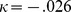 are the best-fit values obtained previously by fitting the decay data for the different strains. The dashed horizontal line indicates the level of 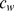 above which within-host fitness is so small that no infection takes place. H5N2 is highlighted as a strain with poor persistence at both low and high temperatures – see text.

So far, we analyzed decay data for specific influenza strains and documented differences in their ability to persist well at low and high temperatures. We can go one step further and study the hypothetical fitness of strains that we did not measure. To do so, we can vary 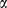 (i.e. clearance rate at 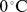) over a wide range of values, and for each value we can compute a corresponding 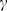 according to the regression equation 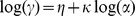 estimated above. We then use the values of 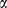 and 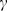 to compute virus decay rate, 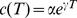 (specifically, 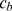 and 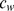 at 5 and 40 degrees Celsius). These values for both the actual virus isolates and the theoretical model are shown in figure 7. The figure shows that not surprisingly, as 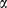 (clearance rate at 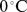) increases, clearance rate 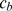 at a close-by low temperature (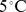) also increases. In contrast, as 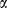 increases (worse low-temperature persistence), the trade-off leads to a decrease of the within-host clearance rate, 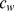, (better high-temperature persistence) – at least initially: At high enough 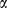, within-host clearance rate starts to increase again. Mathematically, this is due to the fact that at large 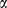 and small 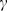, the linear term in the decay equation 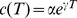 dominates. Biologically, this indicates a strain with poor persistence largely independent of the temperature (i.e. both large 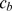 and 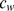). In our dataset, H5N2 seems to fit this description.

**Figure 8:**
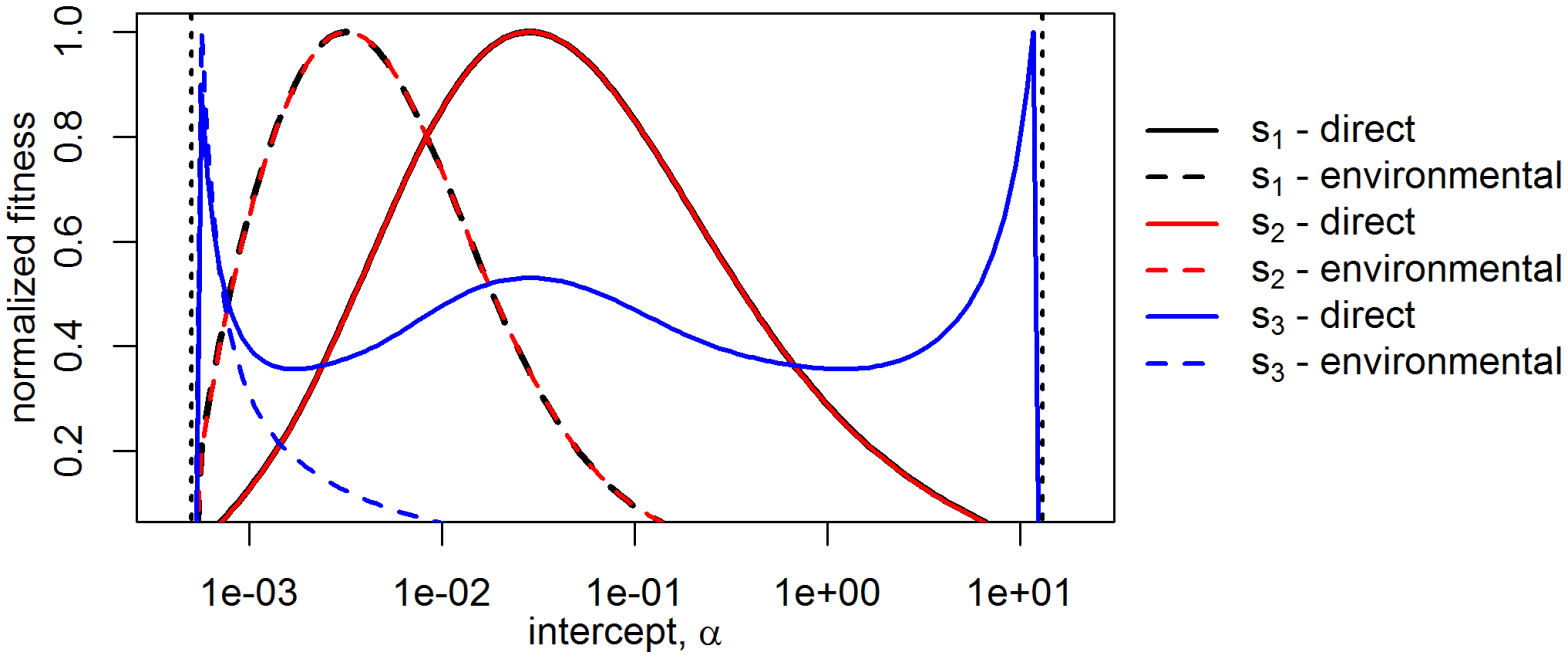
Normalized Fitness as measured by 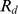 and 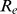 for direct transmission and environmental transmission. The dashed vertical lines indicate the level of 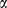 above which 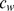 becomes so large that no infection takes place (c.f. horizontal line in figure 7). Note that results for 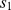 and 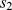 are virtually indistinguishable and therefore the curves are on top of each other.

To determine between-host fitness for a generic strain with given 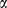 and 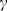 values, we use 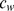 for every value of 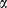 and simulate the within-host infection model, compute duration of infection and total virus load, determine the link functions 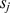, and finally compute fitness as quantified by 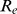 and 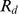. We normalize fitness to 1 to cancel out the different constants of proportionality, as done previously. Figure 8 shows normalized fitness for direct and environmental transmission for the different link functions 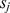. For 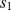 and 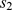, results are virtually indistinguishable. For both 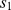 and 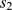, an intermediate level of 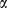 leads to optimal fitness. For direct transmission, with fitness measured by 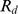, the maximum fitness directly corresponds to the value of 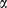 at which 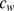 is lowest (see figure 7B). For environmental transmission, the maximum fitness is shifted towards lower 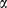 (i.e. lower 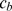) values, meaning better persistence at low temperatures becomes important.

For the scenario where shedding and infection rate are proportional to the logarithm of virus load (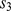) one finds that for environmental transmission, within-host dynamics plays a minor role and persistence at low temperatures (low 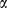) is the dominating component for fitness. Direct transmission for the 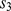 link function produces the most interesting pattern. While this scenario shows a local maximum at medium 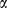 like those seen for 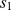 and 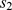 link functions, fitness is highest for either low or high 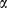, close to the edge at which within-host infection becomes impossible. This is because at those values, virus load is low but the duration of infection is rather long. As explained in the previous section, the long duration of infection can more than make up for the reduced virus levels, leading to an overall increase in 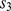 and therefore explaining the high fitness at the edges. The pronounced fitness peaks resulting from long-lasting infections are not seen in a within-host model that includes an immune response (see supplementary materials), and are therefore likely not relevant for influenza in immunocompetent hosts. However, such a long-lasting infection might have relevance for immunocompromised hosts, and is likely important for other pathogens (see e.g. the “sexually transmitted infection regime” in [53]).

## Discussion

Trade-offs between different traits or phenotypes acting at different scales are likely common and have been explored previously (see e.g. [9], [10], [40], [53], [58], [78]–[88] for some recent work). In this study, we focused on trade-offs in temperature-dependent virus decay and analyzed how the interaction of within- and between-host scales determines overall fitness. Taking a panel of influenza A strains, we found evidence that a trade-off exists between the ability to persist at low temperatures versus high temperatures. Of course, the negative correlation found in the data should by no means be taken as proof of the existence of such a trade-off. Further, more detailed studies are needed to investigate this potential trade-off more carefully. If the finding holds up, it would also be very interesting to elucidate the mechanism responsible for this trade-off. As it currently stands, we consider the observed pattern as an interesting suggestion that made it worthwhile to investigate how – given such a trade-off – the within-host and between-host scales interact to impact overall fitness. By linking the within-host dynamics to the population level, we were able to estimate population level-fitness as measured by the reproductive number for both the cases where transmission is through direct contact between birds and where transmission occurs through an environmental stage (i.e. virus persistence in water). We found that if direct transmission is dominant, viruses that persist well at high temperatures and therefore perform well within a host also had the best between-host fitness. This trend was most pronounced if transmission or shedding was directly proportional to the total within-host virus load. For the environmental transmission scenario, the balance was somewhat shifted toward viruses with good environmental, low-temperature, persistence. This was especially true if shedding and infection rate were assumed to be proportional to the logarithm of the virus load.

In the supplementary materials, we also explored the impacts of taking into account an immune response. We found a somewhat diminished importance of differences in the between-host decay rate between strains. This, in turn, leads to greater emphasis on the fitness contribution of environmental persistence. Along similar lines, a brief analysis of a model including virulence suggests that if high within-host fitness leads to host death and thereby interruption of transmission, the balance would be further tipped toward strains that have good environmental persistence.

Both more detailed within-host models including further aspects of the immune response and more detailed virulence models are worthwhile avenues for further studies. So are models with more detailed links of the within-host and between-host scales. However, to go beyond qualitative results, the right kind of data would need to be available to allow proper specification and parameterization of such more complex models.

In addition, it will be worthwhile to follow up with studies that look at virus fitness beyond the reproductive number. Specifically, given the epidemic behavior of influenza, a model that would explicitly simulate multiple rounds of seasonal between-host outbreaks (along the lines of [16]) and track persistence and extinction of strains with different temperature-dependent persistence strategies might be insightful. Similarly, a more detailed model of environmental persistence, e.g. through inclusion of seasonal variation and other dynamical features, and its effect on fitness as measured by the reproductive number or some other suitable quantity might be of interest.

Another fruitful topic for future studies is to investigate additional potential trade-offs. It is known that temperature has an effect on other phenotypes, such as virus binding efficiency or the performance of polymerase. This could be included in a model by making other model parameters temperature-dependent. Provided the right kind of data were available, one could then study how temperature impacts these additional parameters and thereby overall fitness.

In summary, our results show that differences in fitness can at times be substantial and strongly depend on transmission route and how within-host and between-host models are linked. Based on our findings, we predict that if shedding and infection rates are proportional to virus load, virulence is negligible, and within-host virus clearance is primarily determined by temperature-dependent virus decay, there is strong evolutionary pressure for influenza viruses to increase persistence at high temperatures. Conversely, if virus shedding and direct transmission rates scale with the logarithm of virus load, if virulence plays an important role, or if within-host virus clearance is essentially via the immune response or other non-temperature dependent mechanisms, influenza viruses with good environmental persistence at low temperatures should be favored.

## Supporting Information

Figure S1
**Flow diagram for the within-host model with a B-cell/antibody immune response.** 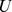, 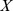, 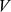 and 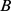 are the variables describing uninfected cells, infected cells, virus and B-cells/antibodies. Uninfected cells become infected at rate 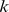, infected cells produce virus at rate 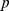 and die at rate 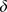. Virus decays at rate 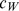. B-cells/antibodies expand exponentially through clonal expansion at rate 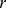 and remove virus at rate 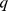. Solid lines indicate physical flows, dashed lines indicate interactions.(TIFF)

Figure S2
**Relative fitness for different strengths of the immune response.** Left column shows direct transmission scenarios, right column shows environmental transmission scenarios. The rows show from top to bottom the different forms of linking within-host virus load to between-host transmission, i.e. 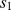, 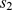, 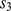. Note that for clarity of representation, we use a linear scale for the direct and a log scale for the environmental transmission scenario.(TIFF)

Figure S3
**Fitness as measured by** 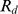 **and** 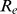 **(normalized to 1) for direct transmission and environmental transmission, with immune response at** 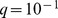 **.** The dashed vertical lines indicate the levels of 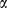 where 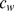 becomes so large that no infection takes place. Note that results for 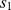 and 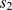 are virtually indistinguishable and therefore the curves are on top of each other.(TIFF)

Figure S4
**Relative fitness for the A) direct and B) environmental transmission scenario for different shedding definitions in the presence of virulence.** Fitness for H6N4 in the environmental transmission scenario with link-function 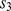 is 50 and not shown on the plot.(TIFF)

Text S1 **Additional Results for a within-model including an immune response and a scenario including virulence.**
(PDF)
