# Supplementary figures and images for: A Multi-scale Analysis of Influenza A Virus Fitness Trade-offs due to Temperature-dependent Virus Persistence
Source: PLoS Comput Biol. 2013 Mar 21;9(3):e1002989. doi: 10.1371/journal.pcbi.1002989 (PMC3605121; doi:10.1371/journal.pcbi.1002989)

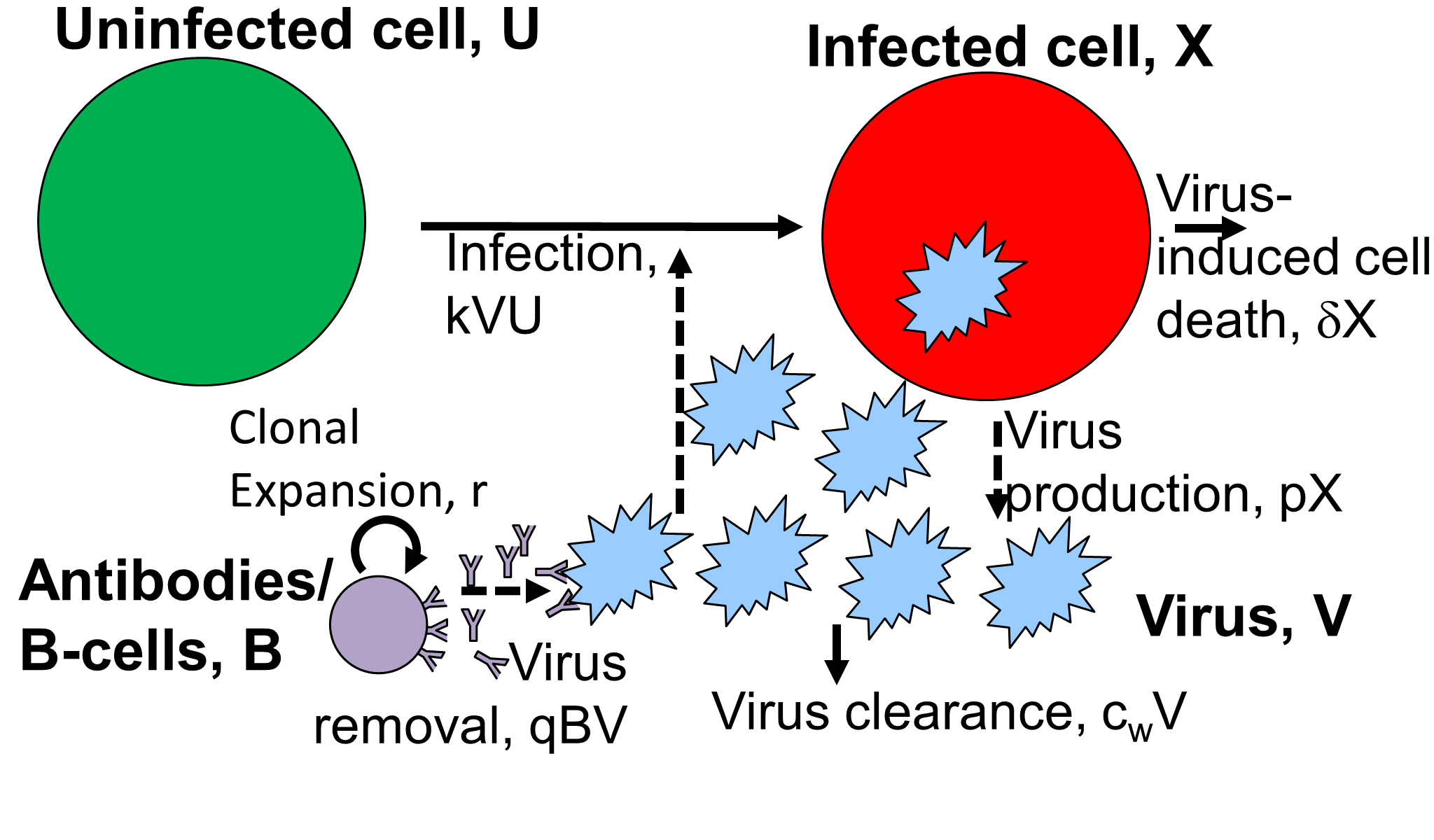

Supplement: Figure S1 — Flow diagram for the within-host model with a B-cell/antibody immune response. , , and are the variables describing uninfected cells, infected cells, virus and B-cells/antibodies. Uninfected cells become infected at rate , infected cells produce virus at rate and die at rate . Virus decays at rate . B-cells/antibodies expand exponentially through clonal expansion at rate and remove virus at rate . Solid lines indicate physical flows, dashed lines indicate interactions. (TIFF) [file pcbi.1002989.s001.tiff]

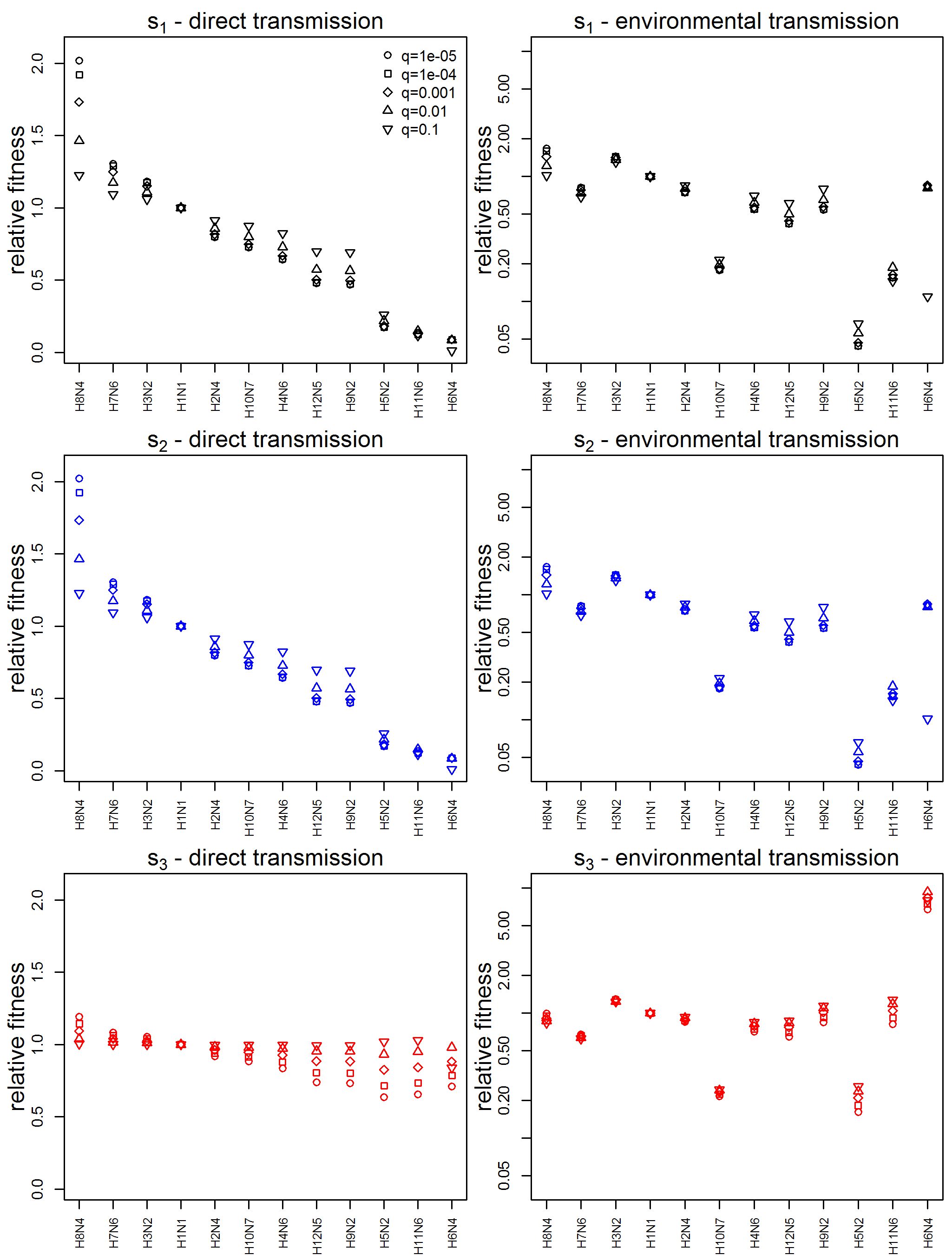

Supplement: Figure S2 — Relative fitness for different strengths of the immune response. Left column shows direct transmission scenarios, right column shows environmental transmission scenarios. The rows show from top to bottom the different forms of linking within-host virus load to between-host transmission, i.e. , , . Note that for clarity of representation, we use a linear scale for the direct and a log scale for the environmental transmission scenario. (TIFF) [file pcbi.1002989.s002.tiff]

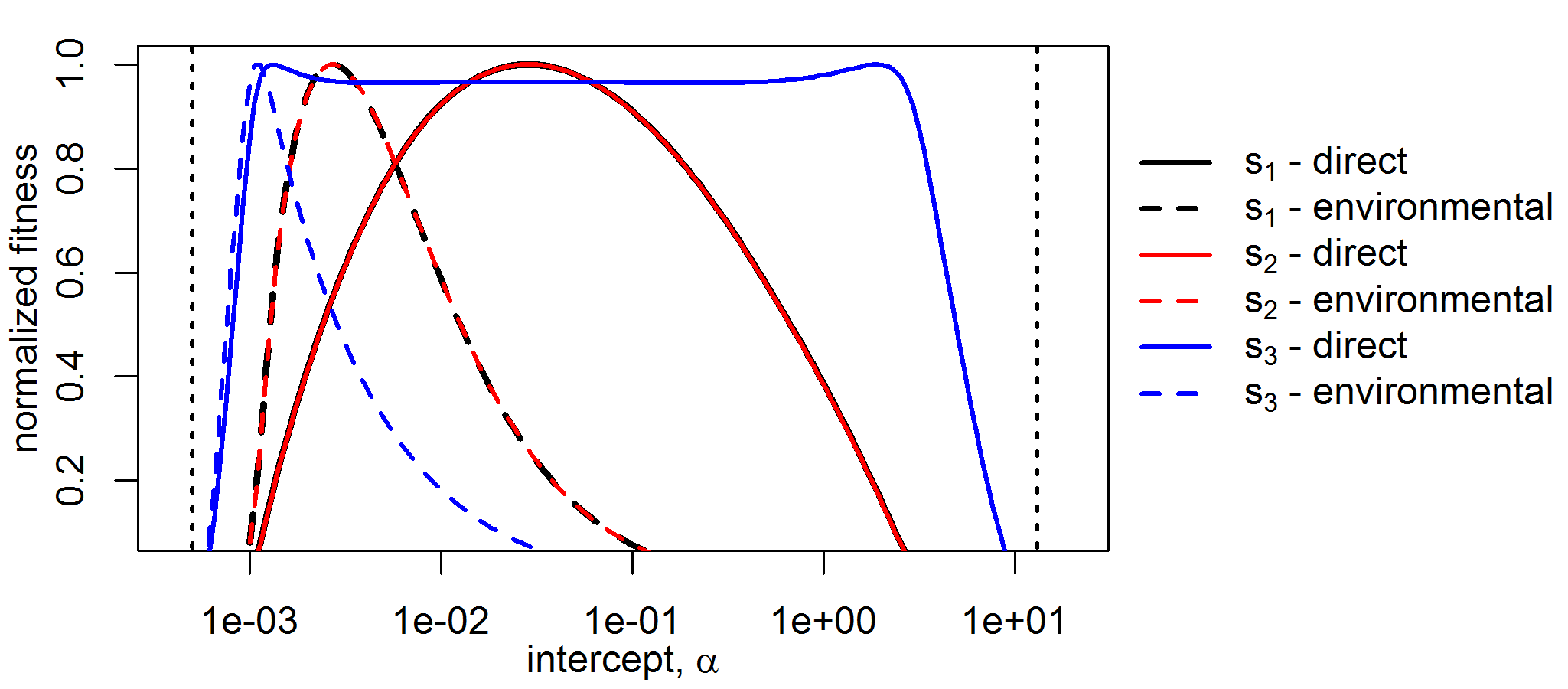

Supplement: Figure S3 — Fitness as measured by and (normalized to 1) for direct transmission and environmental transmission, with immune response at . The dashed vertical lines indicate the levels of where becomes so large that no infection takes place. Note that results for and are virtually indistinguishable and therefore the curves are on top of each other. (TIFF) [file pcbi.1002989.s003.tiff]

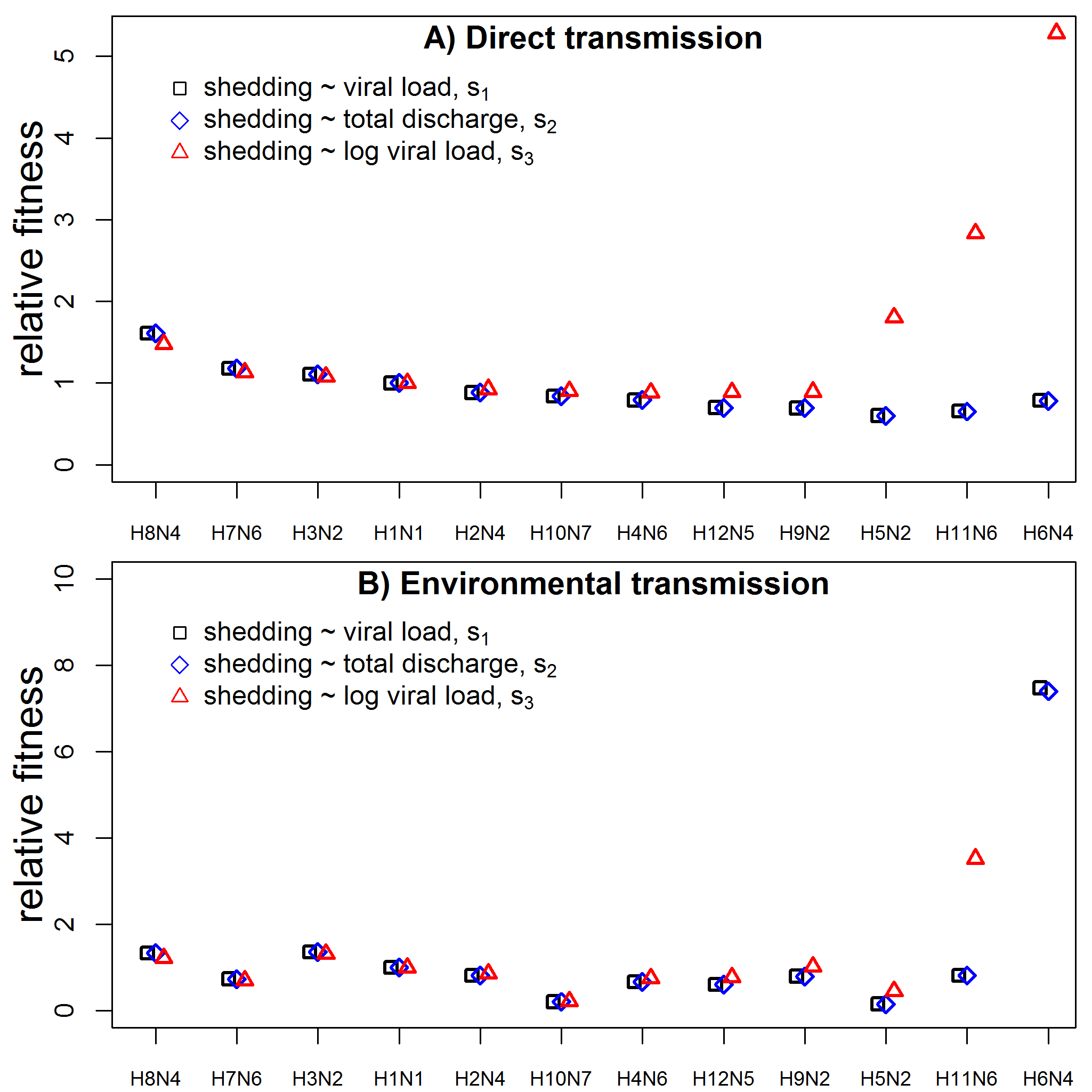

Supplement: Figure S4 — Relative fitness for the A) direct and B) environmental transmission scenario for different shedding definitions in the presence of virulence. Fitness for H6N4 in the environmental transmission scenario with link-function is 50 and not shown on the plot. (TIFF) [file pcbi.1002989.s004.tiff]
