# Supplementary material for: A Multi-scale Analysis of Influenza A Virus Fitness Trade-offs due to Temperature-dependent Virus Persistence
Source: PLoS Comput Biol. 2013 Mar 21;9(3):e1002989. doi: 10.1371/journal.pcbi.1002989 (PMC3605121; doi:10.1371/journal.pcbi.1002989)
Supplement: Text S1 — Additional Results for a within-model including an immune response and a scenario including virulence. (PDF) [file pcbi.1002989.s005.pdf]

Supplementary Text: A multi-scale analysis of influenza A virus fitness trade-offs due to temperature-dependent virus persistence

Andreas Handel[1*], Justin Brown[2], David Stallknecht[2], Pejman Rohani[3,4,5]
1 Department of Epidemiology and Biostatistics, College of Public Health, University of Georgia, Athens, GA 30602, USA
2 Department of Population Health, College of Veterinary Medicine, The University of Georgia, Athens, Georgia 30602, USA
3 Department of Ecology and Evolutionary Biology, University of Michigan, Ann Arbor, MI 48109, USA
4 Center for the Study of Complex Systems, University of Michigan, Ann Arbor, MI 48109 USA
5 Fogarty International Center, National Institutes of Health, Bethesda, MD 20892, USA
∗ Corresponding author: ahandel@uga.edu

A within-host model with an immune response

In the main text, we considered the simplest possible within-host model that can properly describe an acute viral infection. This simple model without an immune response can describe most data for influenza virus infections rather well [1, 2]. However, it is also known that influenza infections activate the immune response, which likely plays some role in viral clearance, though the exact contribution of various components of the immune response to virus clearance is still not fully understood. One prominent mechanism of immune response action is through antibodies, which attach to virus and effectively neutralize it. For our model, this is implemented by an antibody clearance term ($-qBV$) in the virus equation, where B is the strength of the antibody (B-cell) immune response and q is the rate of clearance. For $q = 0$, the model reduces back to the one without an immune response shown in the main text. It is still not known how a detailed, accurate model for the immune response in general and B-cells/antibodies in particular should look like. What is known is that B-cells undergo clonal expansion through cell division, leading to an exponential increase in cell numbers; a concurrent exponential increase in antibody levels is usually observed [3]. We use such a simple, heuristic model here, i.e. we assume exponential growth at rate r for the B-cells/antibodies. More detailed models are possible, see for instance recent work by us and others [2, 4, 5]. While more complicated immune response models tend to be more realistic, those models usually lead to the same exponential increase dynamics and since our focus is not on detailed models of the immune response, the simple exponential growth model is the most parsimonious choice. Also note that since we are only interested in the dynamics until virus clearance, we can ignore the eventual contraction phase for the B-cells/antibodies, which sets in after virus clearance has occurred. The model equations are given by

$$\frac{dU}{dt} = -kUV \qquad \text{uninfected cells} \tag{1}$$
$$\frac{dX}{dt} = kUV - \delta X \qquad \text{infected cells} \tag{2}$$
$$\frac{dV}{dt} = pX - c_w V - qBV, \qquad \text{virus} \tag{3}$$
$$\frac{dB}{dt} = rB \qquad \text{B-cells/antibodies,} \tag{4}$$

The model is illustrated in supplementary figure S1.

The impact of an antibody immune response on fitness

For the results presented in the main text, we assumed that within-host removal of virus at rate c_w is purely due to the same degradation mechanisms as found in the external environment. However, removal of virus within a host likely occurs for a number of reasons, most notably due to antibody-mediated clearance. The relative importance of the different clearance mechanisms is not well known. Intuitively, if clearance of virions inside a host were almost exclusively due to the immune response and not natural degradation, the temperature-dependent within-host decay rate c_w would not matter and every virus should optimize its environmental persistence, i.e. minimize c_b. However, antibodies or other immune response components tend assume greater importance towards the end of an infection [4, 5]. We can explore the potential impact of additional virus clearing mechanisms by investigating how results change as we increase the value of q, i.e. antibody-mediated clearance, in the model with immune response presented in the previous section. We ran the simulations for $q = 10^{-5}$, 10^{-4}, 10^{-3}, 10^{-2} and 10^{-1}, which leads to a fraction of virus cleared due to the immune response (i.e. $\int qBV / \int (c_w + qBV)$) in the ranges of $0.01 - 3.4\%$, $0.14 - 9.2\%$, $1.3 - 23.3\%$, $10.9 - 50.9\%$ and $53.6 - 83.1\%$ for the different strains.

Supplementary figure S2 shows relative fitness for direct (left column) and environmental (right column) transmission and the three link-functions for different strengths of the immune response. One sees that as clearance due to the immune response increases, the differences of c_w between the strains become less important, resulting in decreases of relative fitness for strains with low c_w (e.g. H8N4) and increases in relative fitness of strains with high c_w (e.g. H9N2). This pattern holds for both direct and environmental transmission and the different shedding functions. As expected, the effect is strongest for shedding function s_1 and direct transmission, the situation where differences in c_w have the highest impact on fitness. One interesting feature to note is the reduction of relative fitness for the H6N4 (and to a lesser extent the H11N6) strain at very high immune response strength. This is because at such high levels of q, the within-host dynamics is close to the level where no infection occurs. At this limit, the nonlinear dependence of virus load on c_w is so strong that having a higher c_w greatly reduces virus dynamics in such a way that overall relative fitness shifts again in favor of the low c_w strains. If q is further increased such that other strains are pushed close to a regime where infection becomes impossible, one sees this reversal in fitness for these strains as well. Obviously, at such high q, the H6N4 and H11N6 strains fail to produce any within-host infection and comparison becomes impossible.

As in the main text, we can also investigate how the presence of the immune response alters results for a general trade-off scenario where we vary α (i.e. clearance rate at $0\,^{\circ}\mathrm{C}$) over a wide range of values. Supplementary figure S3 shows normalized fitness over a range of α values for a within-host model with a relatively strong immune response. Once an immune response is present, the infection always ends at around the same time due to immune-response induced clearance (as is observed for influenza in immunocompetent hosts). Comparing this figure to the equivalent one shown in the main text, one sees that this leads to the almost complete disappearance of high fitness at the extremes. In fact, for transmission according to the logarithm of the virus (s_3), fitness is essentially flat for a large range of α values, echoing the finding in supplementary figure S2 where increasing immune response pushed the fitness values for all strains close together.

A model for fitness in the presence of virulence

For the derivation of fitness in the main text, we assumed that the infection does not lead to death. A host is infectious until some time D after infection and is then considered recovered. This is likely a good assumption for low-pathogenic influenza A infections in ducks. However, for high-pathogenic strains, this assumption might not be suitable. If a host were to die due to the infection, it might cut short the

duration of infectiousness and with it potentially the amount of virus that is transmitted or shed. It is still mostly unknown how complex phenotypes, such as morbidity and mortality (virulence) are related to within-host processes of virus dynamics and immune response mediated immunopathology. One possible simple approximation would be to assume that mortality increases with peak or total virus load. Peak pathogen load has been used in previous simple models for virulence [6]. This assumption suggests that while a longer duration of infection and a higher total virus load are beneficial for transmission, a high virus peak is detrimental. A very simple way to capture this idea in our model is to discount the link functions s_j based on virus peak load, i.e. we could define

$$s_j^* = s_j / V^*, \tag{5}$$

where V^* measures virus peak in some suitable way. A linear measure would be the simplest choice, i.e. setting $V^* = V_{\text{peak}}$. However, it is likely biologically more realistic to consider a logarithmic weighting of peak virus load and potential mortality, i.e. $V^* = \log(V_{\text{peak}})$. We consider both measures below. We want to point out the obvious: This is an incredibly simplistic model for virulence. A more thorough study of this aspect would likely require a probabilistic model to look at distributions of outcomes. However, such a more detailed study only makes sense once we better understand how virus and immune response dynamics influence mortality, and is likely not important for most avian influenza infections.

The impact of virulence on fitness

We first tested the assumption that virulence is proportional to a logarithmic measure of peak virus and affects the link function through $s_j / \log(V_{\text{peak}})$. We found that this assumption lead to almost no noticeable differences compared to the previous assumption with no virulence (plots not shown). We then tested the (probably less biologically realistic) model where V^* in equation (5) is given directly by the virus peak instead of the logarithm. Supplementary figure S4 shows that accounting for virulence in this way, strains with poor within-host performance (high c_w) perform relatively better, because now strains with differences in peak virus load have a stronger impact on transmission-reducing virulence, which gives strains with lower within-host virus peak loads and advantage. Overall, this suggests that virulence could have a role in influencing the importance of within-host versus between-host performance. However, absent any solid understanding how within-host virus load and immune response affect virulence, more robust conclusions are not possible.

References

1. Smith AM, Perelson AS (2011) Influenza a virus infection kinetics: quantitative data and models. Wiley Interdiscip Rev Syst Biol Med 3: 429–445.

2. Beauchemin CAA, Handel A (2011) A review of mathematical models of influenza A infections within a host or cell culture: lessons learned and challenges ahead. BMC Public Health 11 Suppl 1: S7.

3. Iwasaki T, Nozima T (1977) Defense mechanisms against primary influenza virus infection in mice. I. The roles of interferon and neutralizing antibodies and thymus dependence of interferon and antibody production. J Immunol 118: 256–263.

4. Miao H, Hollenbaugh JA, Zand MS, Holden-Wiltse J, Mosmann TR, et al. (2010) Quantifying the early immune response and adaptive immune response kinetics in mice infected with influenza a virus. J Virol 84: 6687–6698.

5. Handel A, Longini IM, Antia R (2010) Towards a quantitative understanding of the within-host dynamics of influenza A infections. J R Soc Interface 7: 35–47.

6. Antia R, Levin BR, May RM (1994) Within-host population dynamics and the evolution and maintenance of microparasite virulence. The American Naturalist 144: 457-472.
